# Toll-Like Receptors Expression and Signaling in Glia Cells in Neuro-Amyloidogenic Diseases: Towards Future Therapeutic Application

**DOI:** 10.1155/2010/497987

**Published:** 2010-07-25

**Authors:** Dorit Trudler, Dorit Farfara, Dan Frenkel

**Affiliations:** Department of Neurobiology, George S. Wise Faculty of Life Sciences, Tel Aviv University, Sherman building, Room 424, Tel Aviv 69978, Israel

## Abstract

Toll-like receptors (TLRs) are known to be expressed by innate immune response cells and to play a critical role in their activation against foreign pathogens. It was recently suggested that TLRs have an important role in the crosstalk between neurons and glial cells in the central nervous system (CNS). TLR signaling was reported to be associated with a yin-yang effect in the CNS. While TLR signaling was linked to neurogenesis, it was also found to be involved in the pathogenesis of neurodegenerative diseases. This paper will focus on TLR signaling in glial cells in neurodegenerative diseases such as Alzheimer's disease, prion diseases, amyotrophic lateral sclerosis, and Parkinson's disease. Understanding the pattern of TLR signaling in the glial cells may lead to the identification of new targets for therapeutic application.

## 1. Introduction

Toll-like receptors (TLRs) recognize conserved pathogen-associated molecular patterns (PAMPs) of bacteria, viruses, yeast, fungi, and parasites [1]. At least 13 TLR genes exist in mammals, and functional ligands have been identified [2]. TLRs 1–9 are expressed in both mice and humans, whereas TLRs 10–13 are expressed only in mice [3].

A tight control of the TLR pathway is essential for maintaining homoeostasis, since overactivation of TLRs has been linked to various infectious and inflammatory diseases. TLR engagement leads to the activation of the transcription factor nuclear factor *κ*B (NF-*κ*B), which regulates the induction of proinflammatory cytokines such as tumor necrosis factor *α* (TNF*α*), Interleukin-1*β* (IL-1*β*), and Interleukin-6 (IL-6). It can also activate members of the mitogen-activated protein kinase (MAPK) family including p38 and c-Jun N-terminal kinase (JNK) [3]. These kinases are involved in the transcription of genes and regulate mRNA stability. A tight regulation of these pathways results from post-translational modification processes.

Activation of TLR-mediated signaling by various agonists does not always involve a straightforward lock-and-key mode of ligand-receptor binding. The extracellular domains of all TLRs share important structural features, yet mediate responses to widely different agonists, pointing out that more complex interactions are involved. Many additional proteins are required for the activation of TLR-mediated signaling by their agonists, including coreceptors and docking molecules on the cell surface and binding catalysts that promote certain interactions, such as heat shock proteins [4–6]. In addition to the essential contribution by coreceptors and accessory interaction partners, most TLRs also operate as homo- or heterodimers.

## 2. TLR Signaling Pathways

TLRs are type 1 transmembrane glycoproteins characterized by the presence of a leucine-rich repeat (LRR) domain and a Toll/IL-1 receptor (TIR) domain [7]. LRRs are found in a diverse number of proteins and are involved in ligand recognition and signal transduction [8]. The intracellular TIR domain portion consists of approximately 200 amino acids and contains sequences that are highly conserved among family members. TLRs are proposed to dimerize following ligand binding, resulting in the recruitment of TIR domain-containing adaptor molecules to initiate downstream signaling through interactions within the TIR regions [9].

The family of TLRs share structural properties of not only their extracellular LRR structures but also their intracellular domains, which interact with intracellular adaptor proteins that relay the agonist engagement signal. Currently, five such adaptors are known. The dominant and founding member of this family of adaptors is myeloid differentiation factor 88 (MyD88), which relays the signal for most TLR family members and tends to predominantly induce an NF-*κ*B-mediated activation of genes, including those encoding TNF-*α*, chemokine (C-C motif) ligand 5 (CCL5 or RANTES), IL-1*β*, and chemokine interleukin 8 (IL-8; CXCL8). The less dominant adaptors are MyD88 adaptor-like protein (Mal), TIR domain-containing adaptor inducing interferon-*β* (TRIF), and TRIF-related adaptor protein (TRAM). Recent evidence indicates that TRIF associates with TRAF6 and induces NF-*κ*B signaling toward cytokine and chemokine production [10]. The fifth TIR adaptor SARM (sterile-*α* and HEAT/Armadillo motifs-containing protein) has been shown to inhibit TRIF [11].

TLR signaling may lead to different responses in distinctive cell types through an interaction with MyD88 unique variants. Not every cell expresses the same set of adaptors. For example, it is rapidly becoming clear that a selective expression of the less-frequently used type of MyD88 in neurons, renders these cells uniquely sensitive to TLR-mediated activation of the JNK pathway to apoptosis, instead the NF-*κ*B pathway towards inflammatory response as in glia cells. In this way, selective expression of adaptors strongly influences the quality of the response mounted by different types of cells to a given TLR agonist.

TLR4 was the first TLR to be identified as an orthologue of Drosophila Toll [12, 13]. Structures of TLR2, 3, and 4 with their ligands have been recently elucidated and provide an understanding of ligand-induced activation of TLRs [14–17]. All structures of TLRs bound to their ligands, reveal a common “M”-shaped architecture. The C-termini of the extracellular domains converge, therefore allowing the interaction between TIR domains to occur and initiate downstream signaling events [18].

TLR ligands encompass a broad spectrum of pathogens. Each pathogenic ligand binds to a specific receptor, for example: TLR2 plays an important role in the recognition of fungal, gram-positive, and mycobacterial components. TLR2 can form a complex with TLR1 or TLR6 and respond to lipopeptides from a wide variety of microbes. The TLR1–TLR2 dimer responds to triacylated lipopeptides, whereas TLR2–TLR6 responds to diacylated lipopeptides. TLR3 recognizes double-stranded RNA (dsRNA). TLR4 is responsible for the recognition of lipopolysaccharide (LPS) while TLR5 is responsible for the recognition of bacterial flagellin [19]. Single-stranded RNA is recognized by TLR7 and TLR8, and TLR9 recognizes DNA which can be either host or pathogen derived [3]. TLR9 is associated with the cellular response against bacterial cytosine phosphate guanosine (CpG) DNA [20]. Interestingly, several TLR family members, including TLR2 and TLR6, appear to cooperate in the recognition of different PAMPs in macrophages [21, 22]. For example, the TLR2-mediated response to phenol-soluble modulin is enhanced by TLR6 but inhibited by TLR1, indicating functional interactions between these receptors [23].

## 3. TLR Signaling Mediates Glial Cell Activation in the CNS

Innate immunity in the CNS depends primarily on the functions of glial cells, especially astrocytes and microglia, which are important for the early control of pathogen replication, direct recruitment, and activation of the adaptive immune system required for pathogen recognition and clearance [24].

Under resting conditions, in rodents in vivo, TLRs 1–9 have been detected in the CNS by quantitative real-time PCR, with particularly strong expression of TLR3 [25]. The levels of TLRs in the CNS can be upregulated by viral and bacterial infection, treatment with TLR stimuli, or CNS autoimmunity [25–28], providing a mechanism for amplification of inflammatory responses to pathogens infecting the CNS. Human glial cells do not necessarily display the same TLR profile as rodent glial cells. Immunostaining of cultured microglia and astrocytes for TLR3 and TLR4, revealed two opposite features. Both TLR3 and TLR4 were found exclusively localized in vesicular structures inside microglia and not on the surface of the cells. However, with cultured astrocytes, TLR3 and TLR4 were found only on the cellular surface [26]. This striking difference in subcellular localization of TLRs between microglia and astrocyte may relate to the difference in phagocytic and antigen processing properties of these cells [29–32].

### 3.1. TLR in Microglia

**Figure 1:**
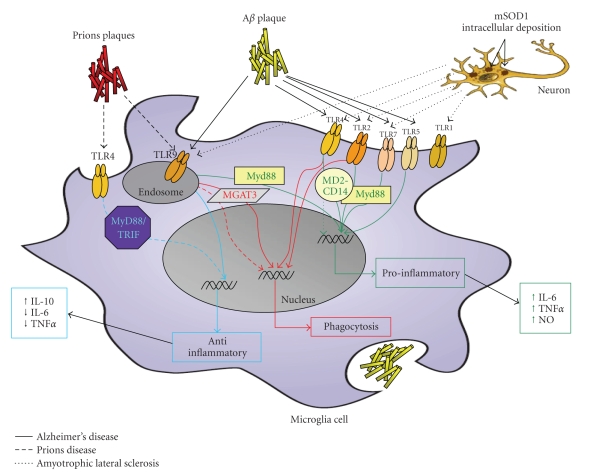
Microglia TLR signaling in neurodegenerative diseases. Abnormal amyloid deposition in different neurodegenerative diseases may activate microglial cells through TLRs. Microglial activation may lead to further neuronal damage through secretion of proinflammatory cytokines (green), such as IL-6 and TNF-*α*, or to neuroprotection by secretion of anti-inflammatory cytokines (blue), such as IL-10, which may prevent further neuronal death. Furthermore, recent reports suggest the role of TLRs 2, 4, and 9 signaling in modulating the phagocytosis (red) and clearance of the neurotoxic amyloid deposition.

Microglial TLRs are crucial as a first line of defence against bacterial or viral infection. In response to the appearance of multiple bacterial or viral TLR agonists, TLR-mediated signaling promotes the production of a variety of inflammatory mediators (reviewed by [33, 34]). In addition, phagocytosis is stimulated by TLR activation, which may be particularly relevant to the clearance of bacteria as well as aggregated or abnormal proteins such as amyloid fibers from the CNS [35, 36] (Figure 1). Like other macrophage-like cells, microglia can express essentially all different TLR family members. While TLR expression is hardly detectable in resting microglia in a healthy CNS, multiple TLRs rapidly appear upon activation of the cells. Primary microglia in vitro constitutively express a wide array of TLRs (TLRs 1–9) at varying levels [26, 37]. Constitutive expression of TLRs is primarily in microglia and largely restricted to the circumventricular organs (CVOs) and meninges, areas with direct access to the circulation, although they may be expressed at lower levels in other regions, too [28, 38, 39]. This unique localization allows the CNS to recognize pathogens which are present in the periphery as well as those that invade the CNS. Like in macrophage, TLRs are exclusively found within endosomal vesicles of microglia, illustrating their primary role in examining the phagocytosed debris. While microglia express all TLRs at readily detectable levels [26], TLRs 1–4 are the most dominant, with TLR2 being the most highly expressed TLR compared to other family members; this applies to microglia in rodents as well as in humans. Lehnard et al. [40] have indicated that microglial cells are the major cell type that expresses TLR4 in the mouse brain.

Exogenous and endogenous TLR ligands activate microglial cells. TLRs may mediate different pathways in microglia leading to either neuroprotective or neurotoxic phenotypes. The activation of microglia with peptidoglycan as TLR2 ligand [35], LPS as agonist for TLR4 [41], or TLR9 ligand CpG [42], markedly boosted the ingestion of Alzheimer's disease neurotoxic Amyloid *β* (A*β*) protein in vitro. A recent report [43] showed an enhanced phagocytic ability of microglia toward amyloid protein as well as cognitive improvement with the administration of a low dose of CpG as an agonist for TLR9. Moreover, this group emphasized that the clearance was not followed by the release of nitric oxide (NO) and glutamate as neurotoxic mediators.

However, activated microglia with TLR ligands also produce neurotoxic molecules such as proinflammatory cytokines, NO, reactive oxygen species (ROS), and peroxynitrite [44]. In particular, LPS-activated microglia produce a large amount of glutamate, an important neurotransmitter but also a potent neurotoxin [45] and LPS injection may activate TLR4 on microglia and is linked to oligodendrocyte injury [40].

### 3.2. TLR in Astrocyte

Primary murine astrocytes express a wide variety of TLRs, but at lower levels than microglia, suggesting that astrocytes may be important for antiviral responses in the CNS. Expression of TLRs 1–9 was found on surface of murine astrocytes. Furthermore, murine astrocyte seems to express under physiological condition high levels of TLR3 [46, 47]. To date, human astrocytes have been reported to express TLRs 1–5 and TLR 9 [26, 48, 49]. The lack of TLRs 6–8 may be due to a difference between species or the result of varying isolation and culture conditions. TLRs 2–4 are clearly detectable on astrocyte in both cell culture models and in vivo during trauma or inflammation [48–50].

TLR signaling in astrocyte can activate the production of a wide range of neuroprotective and anti-inflammatory mediators rather than merely stimulating proinflammatory factors. Like microglia, a healthy human CNS barely expresses TLRs on astrocyte, but once inflammation develops, TLR expression emerges on the cell surface of astrocyte, detectable by immunohistochemistry [26].

The preference of astrocyte to express up to 200-fold elevated levels of TLR3 upon activation is puzzling since the only currently known ligand for TLR3 is dsRNA, which is believed to emerge as an intermediate during viral replication. Yet, dsRNA is generally inside cells rather than secreted into the microenvironment, and its detectable presence in the microenvironment of CNS cells is rare. Bsibsi et al. [48] have demonstrated that the TLR3-mediated response in human astrocyte is far more comprehensive than the TLR4-mediated response. TLR3-mediated activated astrocyte produce a variety of factors that are well-known mediators of both neuroprotection, such as ciliary neurotrophic factor, neurotrophin-4, and vascular endothelial growth factor, and anti-inflammatory cytokines, such as TGF-*β*, IL-10 and IL-11. Indeed, when poly I:C, an agonist for TLR3, is added to organotypic human brain slice cultures, survival of neurons significantly improves [48].

Astrocyte take much longer than microglia to either upregulate TLRs or produce cytokines and growth factors in response to TLR activation. Moreover, TLR3-mediated activation of astrocyte leads to a strong induction of indoleamine 2,3-dioxygenase [50]. This enzyme converts extracellular tryptophan into kynurenine, thereby reducing its concentrations in the microenvironment, which in turn markedly enhances the sensitivity of any nearby T-cell for Fas-ligand-induced apoptosis [51]. In this way, the TLR3-mediated induction of indoleamine 2,3-dioxygenase in astrocyte acts as a local immune-suppressive factor [50].

### 3.3. TLR in Neuronal Cells

Accumulating evidence indicates that TLRs play a role in tissue development, cellular migration, differentiation, and repair processes, especially in response to endogenous molecular ligands. Convincing evidence indicates that neurons can express different functional TLRs, including TLRs 2, 3, 4, and 8 [52–58]. As in other cells, expression levels are dynamic, and influenced by soluble mediators including interferon-*γ*, or by energy deprivation [56, 57, 59]. TLR3 was found to be expressed in cultured human neurons following viral infection [60], and on neurons in human brain tissue in cases of rabies or herpes simplex virus infection [61].

Most TLRs, except TLR3, that are expressed in different cells such as glial cells, signal via the founding family member of the MyD88 family, which predominantly activates an NF-*κ*B-mediated response. The neuronal MyD88 variant, on the other hand, associates with mitochondria, microtubules, and JNK3, and regulates neuronal death during deprivation of oxygen and glucose. Preferred expression of MyD88 in neurons confers a different quality of TLR responsiveness to these cells as compared to cells such as glial cells that do not express this MyD88 variant, but use other adaptors to relay TLR-mediated signaling. As a consequence, TLR3, which is concentrated in the growth cones of neurons, triggers growth cone collapse [55]. TLR2 and 4 induce apoptotic death [54], and TLR8 inhibits neurite outgrowth and triggers apoptosis [53]. In all these mentioned phenomena, signaling pathways operate independently from NF-*κ*B. Clearly, by introducing different MyD88 variants as the dominant adaptor for TLR-mediated intracellular signaling, neurons turn most TLR-mediated signals into negative signals for growth, development, and even survival.

Engagement of TLR4 on neurons induces the expression of nociceptin, an opioid-related neuropeptide [58]. However, this response differs from TLR4-mediated responses in many other cells, in that neurons distinctly use the co-receptor MD-1, instead of the routinely used MD-2, along with CD14 as interaction partners for binding the TLR4 agonist LPS. This illustrates that neurons actually modulate the TLR-signaling platform by not only introducing unusual intracellular adaptors for unique signaling pathways, but also by employing uncommon surface coreceptors, which modulate the response.

## 4. TLR Signaling Link to Neurotoxicity

Although the stimulation of TLRs on glial cells activate functions that are important for the elimination of pathogens, these same functions can be toxic to cells of the CNS that have limited regenerative capacity. LPS exposure causes profound microglial activation associated with oligodendrocyte death, demyelination, and increased vulnerability of neurons to injury, dependent on TLR4 signaling [40, 62]. Similarly, microglia exposed to group B streptococcus (GBS) or *S. pneumoniae* serotype 2 also display neurotoxic properties, dependent on TLR2 [63, 64]. Some reports have demonstrated that LPS-stimulated astrocytes are also neurotoxic, while others have shown that only microglia are required for toxicity [40, 65]. Toxicity appears to be mediated primarily via NO. Indeed, pharmacologic blockade of iNOS is able to prevent neuronal death in the presence of activated glia [64, 65] and to rescue substantia nigra neurons from death [66].

Stimulation of astrocytes with TLR ligands also inhibits their ability to uptake excess glutamate [65, 67], and therefore the role of astrocyte in neurotoxicity may be more critical in models in which glutamate excitotoxicity is a major mechanism of death.

Injection of poly I:C or Pam_3_CysSK_4_ into the CNS can cause neurodegeneration in a TLR3- or TLR2-dependent manner, respectively [63, 68]. Local injections of LPS directly into the CNS cause severe loss of dopamine neurons in the substantia nigra [69] and neurons in the hippocampus [70]. Newborn neurons in the hippocampus and dopaminergic neurons in the substantia nigra appear to be extremely sensitive to the effects of LPS, as peripheral injection of even low levels of LPS reduces the number of these cells [71, 72].

## 5. TLR Signaling Link to Neurogenesis

Neurogenesis is the process by which new neurons are created from neural progenitor cells in the adult brain. It occurs in two major brain regions—the subventricular zone (SVZ) and the dentate gyrus (DG) of the hippocampus [73]. The mechanisms for neurogenesis are emerging with time. Recent evidence suggest that neural progenitor cells also express TLRs [53, 74]. Rolls et al. [74] have proposed that since TLR2 is widely expressed in the brain, and in cells that express early neuronal markers, they may be involved in adult hippocampal neurogenesis. They have demonstrated that in TLR2-deficient mice, there is a reduction in the differentiation of neural progenitor cells into neurons, and an increase in the differentiation into astrocytic cells, and that TLR2 activators increased differentiation. The increase in differentiation was mediated through the NF-*κ*B pathway. In addition, TLR4 has been found to be involved in proliferation via both MyD88-dependent and MyD88-independent pathways. A TLR4 deficiency caused an increase in proliferation and differentiation [74].

TLR8 is dynamically expressed during mouse brain development and localizes to neurons and axons where it may regulate neurite outgrowth and apoptosis [53]. TLR2 and TLR4 are expressed in adult neural progenitor cells and may influence the proliferation and differentiation [74]. Some TLRs are strongly expressed in the embryonic brain and TLR3 and TLR8 have been implicated in neurogenesis and neurite outgrowth in the developing brain whereas TLR2 and TLR4 have been shown to regulate adult neurogenesis [75, 76].

In embryonic development, TLR3 negatively regulates neural progenitor cell differentiation [77]. It has also been suggested recently that in the adult brain TLR3 mediates the production of anti-inflammatory and neuroprotective factors, and thus a TLR3 activation may promote cell survival [48]. TLR8 is a suppressor of murine neurite outgrowth and induces neuronal apoptosis, through an NF-*κ*B independent mechanism. [59].

## 6. TLR Signaling in Neurodegenerative Diseases

A dysfunction of glial and neuronal receptors, which alter the cells sense of their environment, can lead to neurodegenerative diseases. Therefore, the role of TLRs in mediating cell response to stress conditions may play a crucial role in neurological disease progression, as can age-related changes in cell division. Targeting specific TLR signaling may allow the maturation and function of glial and neuronal cells to aid in neuronal repair.

### 6.1. Alzheimer's Disease

Alzheimer's disease (AD) is a progressive neurodegenerative disease, characterized by progressive memory deficits, cognitive impairments, and personality changes. The histopathological hallmarks include deposition of A*β*, neurofibrillary tangles (NFTs), progressive synaptic dysfunction and, much later, neuronal death, especially in the hippocampus [78–80].

While the role of inflammation in disease progression in AD is not fully understood, an increased amount of findings suggest that A*β* deposition and NFT activate a potentially pathological innate immune response in the disease [81]. A*β* plaques are surrounded and infiltrated by activated astrocytes and microglia, which are believed to be the major source of local inflammatory components [82].

An increasing amount of data is emerging that describes the involvement of TLRs in the pathogenesis of AD. The first line of evidence shows an increased expression and upregulation of different TLR genes and TLR-related genes in AD patients and mouse models. For example, the expression level of TLR2 and TLR7 is higher in APP transgenic mice, which accumulate A*β* deposits in their brain, then their matched controls, at 6 months of age [83].

An examination of TLR expression in the brain revealed that there was increased expression of CD14, TLR2, and TLR4 in AD human brains and animal models [41, 84–86]. Plaque-associated microglia exhibit elevated mRNA levels for TLR2, 4, 5, 7, 9 [87] (Figure 1). An injection of A*β* into the hippocampus provokes TLR2 gene expression [88]. It is of interest that a polymorphism in TLR4, which results in a blunted signaling response corresponds to a 2.7-fold reduction in risk for late-onset AD [89].

Fiala et al. [90] have reported that upon A*β* stimulation, mononuclear cells of normal subjects up-regulate the transcription of *β*-1,4-mannosyl-glycoprotein 4-*β*-N-acetylglucosaminyltransferase (MGAT3) (Figure 1). The downstream effect of MGAT3 on phagocytosis may depend upon TLRs and indeed, there was an upregulation following TLRs activation. Interestingly, mononuclear cells of AD patients generally have downregulated MGAT3 and TLR genes as compared to normal individuals. Furthermore, a defective phagocytosis of A*β* may be related to the downregulation of MGAT3, as suggested by an inhibition of phagocytosis using MGAT3 siRNA [90].

Another line of evidence suggests that in AD there is a form of TLR dysfunction that appears in TLR4, which is localized on the surface of microglial cells (Figure 1). A loss-of-function mutation in TLR4 inhibits microglial cell activation towards A*β* depositions, which results in a decrease in the amount of proinflammatory cytokines IL-6 and TNF-*α* and NO. TLR4 has also been found to contribute to A*β*-induced microglia neurotoxicity. A tri-molecular complex consisting of TLR4, MD-2, and CD14 has to be complete for full cellular stimulation by aggregated A*β*. There is also an elevated level of TLR4 in transgenic APP mice and in the brains of AD patients. [86].

Tahara et al. [36] have shown that TLR4 loss-of-function mutation in APP transgenic mice increase diffuse and fibrillar A*β* deposition without an increased expression of APP, and that uptake of A*β* is reduced in TLR4 mutant microglia. They have also demonstrated that the activation of TLR 2, 4, and 9 increased clearance of A*β* [36, 42]. Balistreri et al. reported that a TLR4 polymorphism is involved in aging, and in some age-related diseases such as AD [91]. The phenotypes are associated with changes in cytokine expression. One such haplotype has reduced the production of proinflammatory cytokines [92]. AD patients in the Italian population had an increased frequency of the proinflammatory haplotype [91].

Lotz et al. [93] showed that coadministration of A*β* 1–40 with TLR2 or TLR4 agonists, Pam3-cys and LPS, respectively, led to an additive release of NO and TNF-*α*. However, coadministration of A*β* 1–40 with TLR9 agonist CpG, led to a decrease in the release of NO and TNF-*α*. This suggests that not all TLR agonists enhance the stimulatory effect of A*β* on innate immunity [93]. In microglia (Figure 1), the TLRs functionally interact with other cell surface receptors, including CD36, *α*_6_ *β*_1_ integrin, CD47, and scavenger receptor A, which bind to fibrillar A*β*, to initiate the activation of intracellular signaling pathways [94, 95].

#### 6.1.1. Targeting TLR as Therapeutic Application in AD

APP mouse models with a TLR4 deficiency have an increase in insoluble A*β* in the cortex, as compared to TLR4 wild-type APP mouse models [36]. Thus, factors that increase the microglial cell clearance of A*β*, without producing inflammatory mediators, are candidates for the treatment of AD (Figure 1).

These results suggest that the TLR signaling pathways may be involved in the clearance of A*β* deposits in the brain and that TLRs can be a therapeutic target for application in AD [36]. Indeed, it was shown that an injection to the intrahippocampus of LPS derivatives (a TLR4 ligand) to the brains of AD mice reduced A*β* load, suggesting that the activation of microglia by TLR4 may be therapeutic in AD [96].

Bisdemethoxycurcumin is a natural curcumin, a minor constituent of turmeric (curry), that enhances phagocytosis and the clearance of A*β* in cells from most AD patients, and increases transcription of the MGAT and TLR genes [97]. Furthermore, administration of CpG, a TLR9 activator, in APP transgenic mice, resulted in clearance of A*β* from microglial cells [42].

In conclusion, TLR activation may modulate glial cell activity in AD. Recent research suggests the involvement of TLRs 2, 4, 5, 7, and 9 in the proinflammatory response of microglia toward A*β*, which may be linked to neurotoxicity (Figure 1). Nevertheless, the activation of TLRs 2, 4, and 9 were also linked to both phagocytosis of the neurotoxic A*β* and to an anti-inflammatory response (TLR9), which may lead to neuroprotection (Figure 1). Therefore, elevated expression levels of TLRs 2, 4, and 9, through genetic modification or toward specific agonists, may be a therapeutic application in AD. Indeed, a recent publication [98] suggests the use of TLRs 2 and 4 agonist as a specific macrophage activator to increase the clearance of A*β* in an AD mouse model. An alternative therapeutic approach may be the reduction of TLR5 and 7, by using shRNA or specific antagonists (Figure 1).

### 6.2. Prion Diseases

Prions are infectious particles that are composed mainly of proteins. In prion diseases, prions create extracellular aggregates of beta-sheet-rich, misfolded form, in different tissues, such as the spleen, muscles, and brain. In the brain, aggregated prions are presented by neurons, followed by neurodegeneration. Prion diseases are characterized by their transmissibility and are therefore also termed transmissible spongiform encephalopathies (TSE). Prion diseases have occurred in humans and animals for many years. The human prion disease is Creutzfeldt-Jakob disease (CJD) [99]. All known prion disease affect the structure of the brain with neuropathological features such as neuronal loss, astrocytic activation (gliosis), and spongiform change, and all are currently untreatable and fatal [100].

Prions have a role in the activation of the innate immunity, which suggests functional and structural similarities with Drosophila Toll receptors [101]. The mice with mutated TLR4, wherein signaling is prevented, developed Prion disease in a shorter period of time than control mice, but did not exhibit different levels of prions. This indicates an involvement of TLR4 in the progression of the disease [102]. In addition, in human patients with CJD, there is an elevated level of IL-10, which has been suggested to have a protective role in the disease. TLR4 signaling induces IL-10 production, and this may be the pathway by which TLR4 dysfunction may mediate the rapid progression of the disease [103, 104]. Nevertheless, MyD88 knockout mice inoculated with prions have not shown different prion pathogenesis kinetics from the control mice, suggesting that TLRs 1, 2, 6, and 9 signal through the MyD88 pathway and are not involved in the progression of the disease [105, 106] (Figure 1).

#### 6.2.1. Targeting TLR as Therapeutic Application in Prion Diseases

It has been suggested that TLR9 expression may be linked to the progression of prion diseases. Furthermore, treatment with synthetic oligodeoxynucleotides that contain cytosine phosphate guanosine (CpG-ODN) motifs, known to bind to TLR9, have been suggested as possible treatment for prion diseases in a mouse model, by delaying the disease onset [105, 107]. Another explanation may be the effect of CpG-ODN on microglia activation that may lead to prion degradation [108] (Figure 1). Furthermore, as the activation of TLRs in other amyloidogenic diseases, such as AD, has been linked to the clearance of neurotoxic amyloid, it may prove to be a potential therapeutic approach to the prion diseases.

### 6.3. Amyotrophic Lateral Sclerosis

Amyotrophic lateral sclerosis (ALS) is a devastating and chronic neurodegenerative disease, characterized by selective loss of lower and upper motor neurons [109]. Ten percent of ALS cases are familial (fALS) with 20–25% of these cases resulting from various mutations in the SOD1 gene [110]. The toxicity of the mutant SOD1 (mSOD1) is again of function, because mice that lack the gene do not develop the disease [111], and may be a noncell-autonomous progression [112]. One proposed mechanism for pathogenesis is the aberrant oligomerization of mSOD1 proteins in beta-sheet form, that can be stained by thioflavin S [113].

Several studies have demonstrated the involvement of microglia in ALS pathogenesis (Figure 1). For example, microglia with mSOD1 release more superoxide, nitrate and nitrite and induce more neuronal death [114]. It has also been demonstrated that mSOD1 mice had an elevated level of TLR1, 2, 7 and 9 at 8 months of age, as compared to the matched age control group [83]. Treatment of mSOD1 mice with wild-type microglia improved the pathogenesis [114].

Kang and Rivest [115] have demonstrated that mSOD1 activates microglia through the MyD88-dependent pathway, and mice that were transplanted with bone marrow from MyD88 knockout mice exhibited earlier disease onset and a shorter life span. This suggests a crucial effect of MyD88 in an ALS mouse model. Nevertheless, there was no difference in the disease onset and life span between mSOD mice with MyD88 knockout and mice with normal MyD88. However, MyD88 knockout mice had a more activated microglia at the end stage of the disease, and they lost more motor neurons, which suggests that the context of MyD88 deficiency is linked to neurotoxicity.

Zhao et al. [116] have demonstrated that mSOD1 binds to CD14, which has a role in the activation and toxicity of microglia treated with extracellular mSOD1. CD14 is a co-receptor of TLRs 2 and 4, and blocking the signaling of both of these TLRs inhibited microglial activation following extracellular mSOD1 administration. However, they have found that CD14 knockout mice showed a similar disease progression profile as the control mice.

Nguyen et al. [117] activated the innate immune response in mSOD1 mice. They injected systemic LPS, which increased TLR2 expression across the brain and spinal cord in both wild-type and mSOD1 mice, without changing mSOD expression. Chronic systemic administration of LPS exacerbated disease progression and motor neuron degeneration, which shortened life span. The degree of TLR2 elevation showed a correlation to motor neuron degeneration.

#### 6.3.1. Targeting TLR as a Therapeutic Application in Amyotrophic Lateral Sclerosis

While some research suggests TLR signaling has an important role in neurotoxicity in ALS, there is no clear evidence for a specific TLR that may mediate this effect. Further research should aim to distinguish between elevated expression of different TLRs in modulating an inflammatory response in ALS and their role in disease progression. A potential link between TLR signaling and an increase in neurotrophic factor secretion from glial cells may prove to be a therapeutic approach in ALS.

### 6.4. Parkinson's Disease

Parkinson's disease (PD) is a progressive neurodegenerative disorder characterized by resting tremor, muscular rigidity, and gait disturbances [118, 119]. PD is pathologically characterized by the progressive loss of dopaminergic neurons in the substantia nigra pars compacta and their termini in the dorsal striatum [120]. The pathological hallmark of PD is the presence of deposits of aggregated *α*-synuclein in intracellular inclusions known as Lewy bodies [121, 122].

One of the Parkinson's disease animal models is composed of an intranigral LPS injection, which stimulates dopaminergic cell death [123]. In this model, microglial cells are activated, and demonstrate an upregulation of proinflammatory cytokines and free radical production [124, 125]. LPS is a TLR4 activator, which suggests that there may be a TLR involvement in the pathogenesis of PD. In an MPTP mouse model of PD there was increased expression of TLR4 and CD14, suggesting an involvement of the TLR pathway in the pathogenesis of PD [126].

#### 6.4.1. Targeting TLR as Therapeutic Application in Parkinson's Disease

A recent paper showed that *α*-synuclein immunization in a PD animal model may ameliorate disease progression [127]. Targeting mechanisms in which *α*-synuclein activates TLR signaling, may open a new horizon for therapeutic application in PD.

## 7. Conclusion

TLRs play an essential role in modulating self-defense in different types of species: from fly to human. More recently, it has been suggested that TLRs are important to both cell development and cell-cell interaction. The complex of TLR cascade may trigger specific pathways, which affect the fate of cell activation. The CNS is monitored by the BBB from the peripheral immune response, and is dependent on glia surveying for innate immunity. Abnormal amyloid depositions in the CNS may mimic viral or bacterial infection, which may trigger glial cell activation through TLRs. Investigating the neurotoxic and neuroprotective mechanisms of TLR signaling in glial cells may be crucial for understanding their role in the pathogenesis of neurodegenerative diseases, and may pave the route for future therapeutic intervention. Currently, targeting TLRs is being used in different experimental settings, from animal model experiments to clinical trials, including several diseases, such as chronic lung disease and cancer [128]. However, there are very few tests regarding neurodegenerative diseases. We suggest that targeting TLRs and TLR pathways may also be applicable as a possible treatment for those diseases.

## Abbreviations

TLR: Toll-like receptor
CNS: Central nervous system
PAMP: Pathogen-associated molecular pattern
NF-*κ*B: Nuclear factor *κ*B
TNF*α*: Tumor necrosis factor *α*
IL-1*β*: Interleukin-1*β*
IL-6: Interleukin-6
MAPK: Mitogen-activated protein kinase
JNK: c-Jun N-terminal kinase
TIR: Toll/IL-1 receptor
MyD88: Myeloid differentiation factor 88
CCL5: Chemokine (C-C motif) ligand 5
CXCL8: Chemokine interleukin 8
Mal: MyD88 adaptor-like protein
TRIF: TIR domain-containing adaptor inducing interferon-*β*
TRAM: TRIF-related adaptor protein
LPS: Lipopolysaccharide
dsRNA: Double-stranded RNA
CVO: Circumventricular organ
NO: Nitric oxide
ROS: Reactive oxygen species
GBS: Group B streptococcus
AD: Alzheimer's disease
NFT: Neurofibrillary tangles
A*β*: Amyloid *β*
MGAT3: *β*-1,4-mannosyl-glycoprotein 4-*β*-N-acetylglucosaminyltransferase
TSE: Transmissible spongiform encephalopathies
CJD: Creutzfeldt-Jakob disease
CpG: Cytosine phosphate guanosine.
